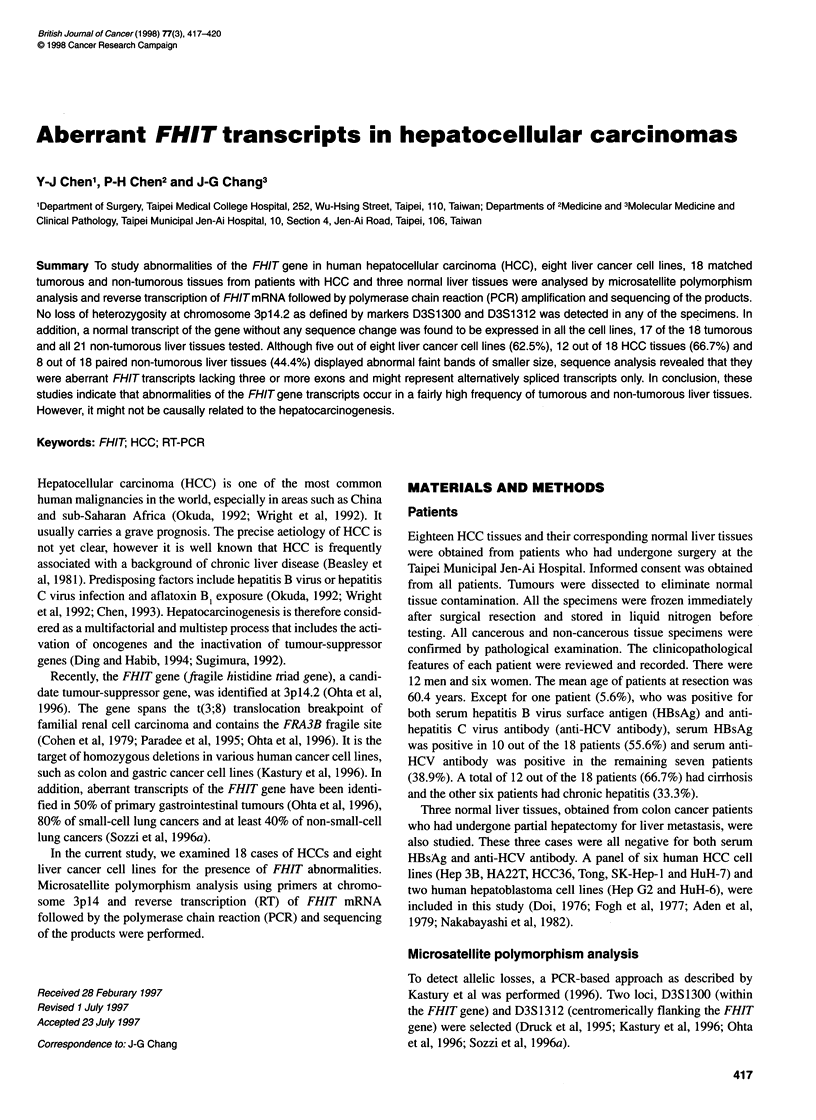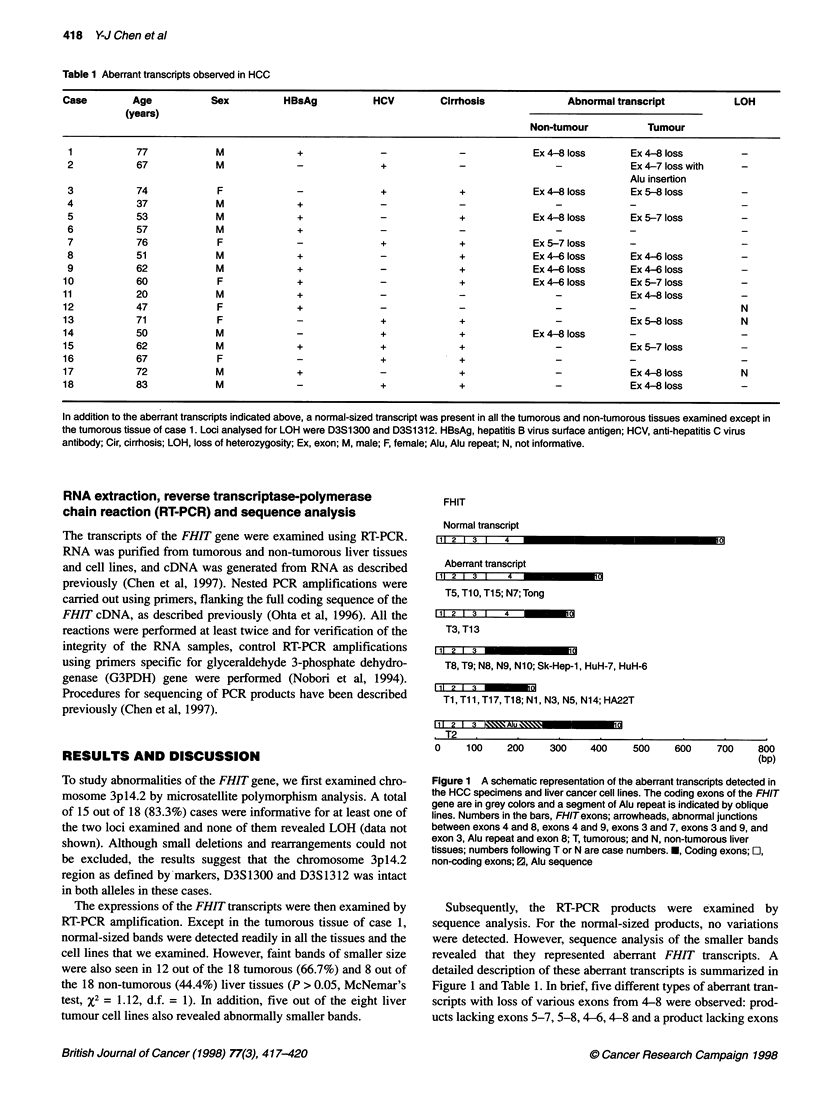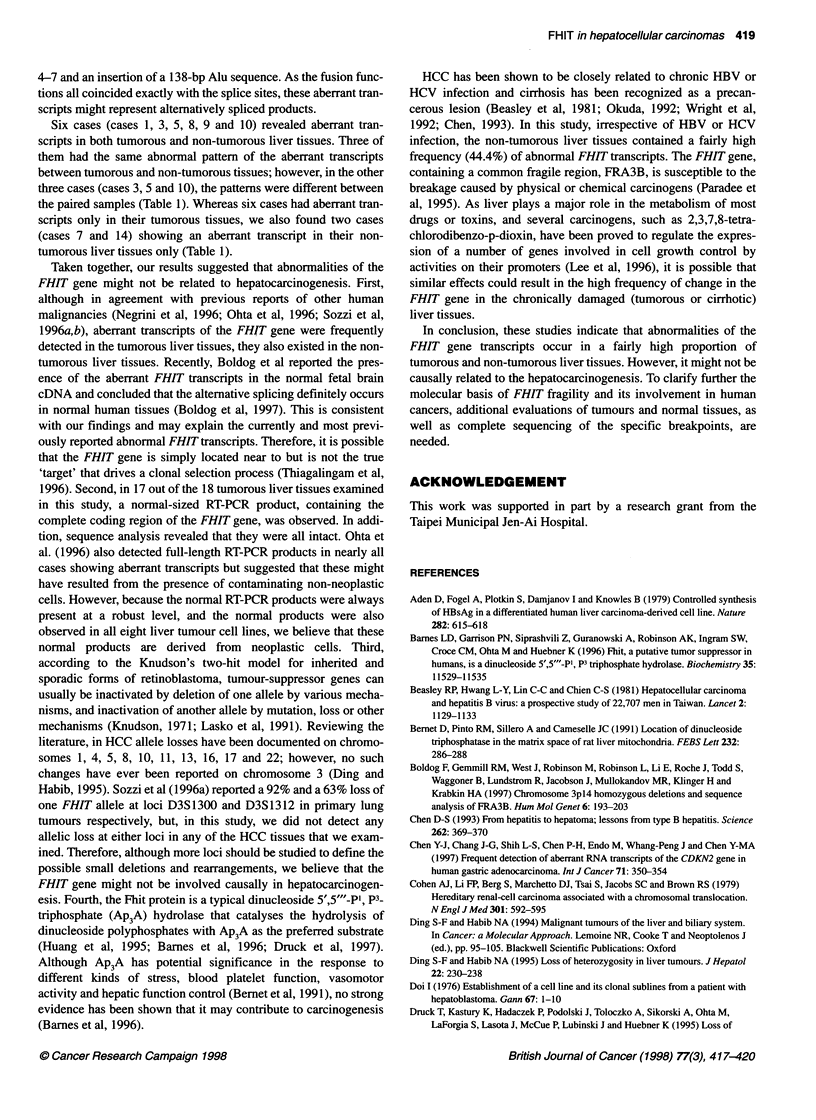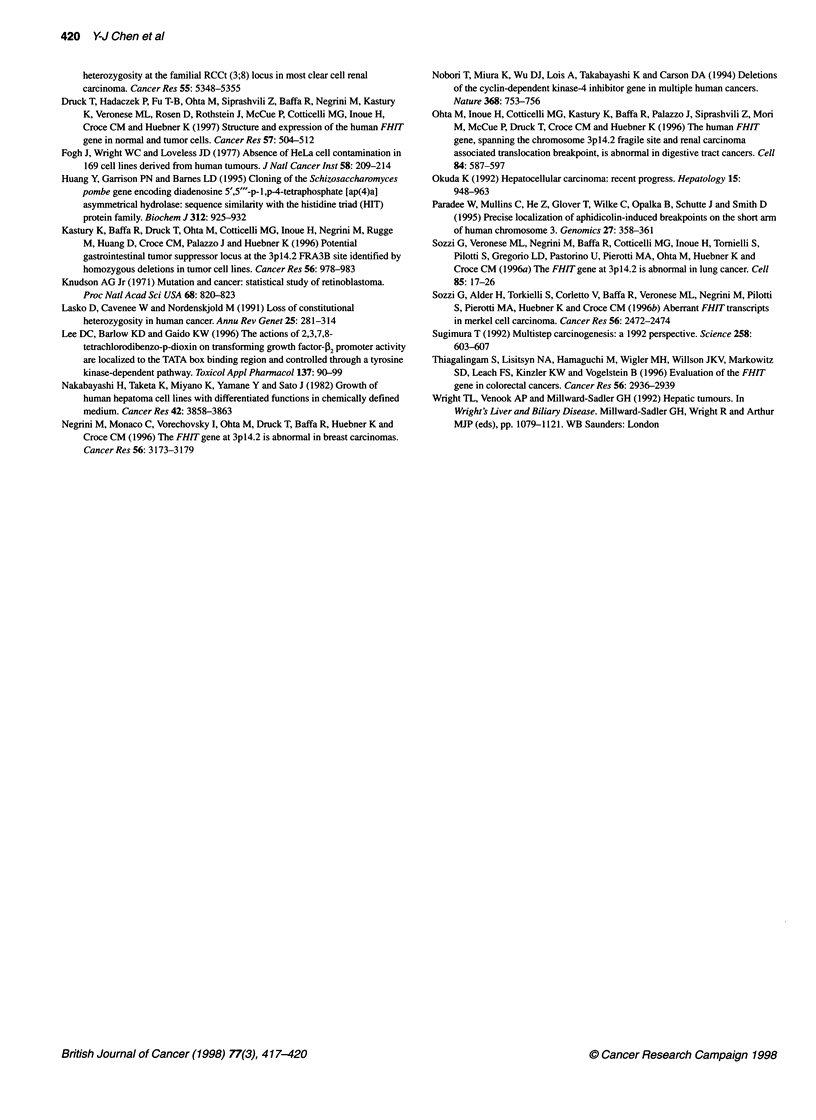# Aberrant FHIT transcripts in hepatocellular carcinomas.

**DOI:** 10.1038/bjc.1998.66

**Published:** 1998

**Authors:** Y. J. Chen, P. H. Chen, J. G. Chang

**Affiliations:** Department of Surgery, Taipei Medical College Hospital, Taiwan.

## Abstract

To study abnormalities of the FHIT gene in human hepatocellular carcinoma (HCC), eight liver cancer cell lines, 18 matched tumorous and non-tumorous tissues from patients with HCC and three normal liver tissues were analysed by microsatellite polymorphism analysis and reverse transcription of FHIT mRNA followed by polymerase chain reaction (PCR) amplification and sequencing of the products. No loss of heterozygosity at chromosome 3p14.2 as defined by markers D3S1300 and D3S1312 was detected in any of the specimens. In addition, a normal transcript of the gene without any sequence change was found to be expressed in all the cell lines, 17 of the 18 tumorous and all 21 non-tumorous liver tissues tested. Although five out of eight liver cancer cell lines (62.5%), 12 out of 18 HCC tissues (66.7%) and 8 out of 18 paired non-tumorous liver tissues (44.4%) displayed abnormal faint bands of smaller size, sequence analysis revealed that they were aberrant FHIT transcripts lacking three or more exons and might represent alternatively spliced transcripts only. In conclusion, these studies indicate that abnormalities of the FHIT gene transcripts occur in a fairly high frequency of tumorous and non-tumorous liver tissues. However, it might not be causally related to the hepatocarcinogenesis.


British Journal of Cancer (1998) 77(3), 417–420
© 1998 Cancer Research Campaign

### Aberrant *FHIT* transcripts in hepatocellular carcinomas

**Y-J Chen[1], P-H Chen[2] and J-G Chang[3]**

[1]Department of Surgery, Taipei Medical College Hospital, 252, Wu-Hsing Street, Taipei, 110, Taiwan; Departments of [2]Medicine and [3]Molecular Medicine and Clinical Pathology, Taipei Municipal Jen-Ai Hospital, 10, Section 4, Jen-Ai Road, Taipei, 106, Taiwan

**Summary** To study abnormalities of the *FHIT* gene in human hepatocellular carcinoma (HCC), eight liver cancer cell lines, 18 matched tumorous and non-tumorous tissues from patients with HCC and three normal liver tissues were analysed by microsatellite polymorphism analysis and reverse transcription of *FHIT* mRNA followed by polymerase chain reaction (PCR) amplification and sequencing of the products. No loss of heterozygosity at chromosome 3p14.2 as defined by markers D3S1300 and D3S1312 was detected in any of the specimens. In addition, a normal transcript of the gene without any sequence change was found to be expressed in all the cell lines, 17 of the 18 tumorous and all 21 non-tumorous liver tissues tested. Although five out of eight liver cancer cell lines (62.5%), 12 out of 18 HCC tissues (66.7%) and 8 out of 18 paired non-tumorous liver tissues (44.4%) displayed abnormal faint bands of smaller size, sequence analysis revealed that they were aberrant *FHIT* transcripts lacking three or more exons and might represent alternatively spliced transcripts only. In conclusion, these studies indicate that abnormalities of the *FHIT* gene transcripts occur in a fairly high frequency of tumorous and non-tumorous liver tissues. However, it might not be causally related to the hepatocarcinogenesis.

**Keywords:** *FHIT*; HCC; RT-PCR

Hepatocellular carcinoma (HCC) is one of the most common human malignancies in the world, especially in areas such as China and sub-Saharan Africa (Okuda, 1992; Wright et al, 1992). It usually carries a grave prognosis. The precise aetiology of HCC is not yet clear, however it is well known that HCC is frequently associated with a background of chronic liver disease (Beasley et al, 1981). Predisposing factors include hepatitis B virus or hepatitis C virus infection and aflatoxin $B_1$ exposure (Okuda, 1992; Wright et al, 1992; Chen, 1993). Hepatocarcinogenesis is therefore considered as a multifactorial and multistep process that includes the activation of oncogenes and the inactivation of tumour-suppressor genes (Ding and Habib, 1994; Sugimura, 1992).

Recently, the *FHIT* gene (*f*ragile *h*istidine *t*riad *g*ene), a candidate tumour-suppressor gene, was identified at 3p14.2 (Ohta et al, 1996). The gene spans the t(3;8) translocation breakpoint of familial renal cell carcinoma and contains the *FRA3B* fragile site (Cohen et al, 1979; Paradee et al, 1995; Ohta et al, 1996). It is the target of homozygous deletions in various human cancer cell lines, such as colon and gastric cancer cell lines (Kastury et al, 1996). In addition, aberrant transcripts of the *FHIT* gene have been identified in 50% of primary gastrointestinal tumours (Ohta et al, 1996), 80% of small-cell lung cancers and at least 40% of non-small-cell lung cancers (Sozzi et al, 1996*a*).

In the current study, we examined 18 cases of HCCs and eight liver cancer cell lines for the presence of *FHIT* abnormalities. Microsatellite polymorphism analysis using primers at chromosome 3p14 and reverse transcription (RT) of *FHIT* mRNA followed by the polymerase chain reaction (PCR) and sequencing of the products were performed.

*Received 28 Feburary 1997*
*Revised 1 July 1997*
*Accepted 23 July 1997*

*Correspondence to:* J-G Chang

### MATERIALS AND METHODS

### Patients

Eighteen HCC tissues and their corresponding normal liver tissues were obtained from patients who had undergone surgery at the Taipei Municipal Jen-Ai Hospital. Informed consent was obtained from all patients. Tumours were dissected to eliminate normal tissue contamination. All the specimens were frozen immediately after surgical resection and stored in liquid nitrogen before testing. All cancerous and non-cancerous tissue specimens were confirmed by pathological examination. The clinicopathological features of each patient were reviewed and recorded. There were 12 men and six women. The mean age of patients at resection was 60.4 years. Except for one patient (5.6%), who was positive for both serum hepatitis B virus surface antigen (HBsAg) and anti-hepatitis C virus antibody (anti-HCV antibody), serum HBsAg was positive in 10 out of the 18 patients (55.6%) and serum anti-HCV antibody was positive in the remaining seven patients (38.9%). A total of 12 out of the 18 patients (66.7%) had cirrhosis and the other six patients had chronic hepatitis (33.3%).

Three normal liver tissues, obtained from colon cancer patients who had undergone partial hepatectomy for liver metastasis, were also studied. These three cases were all negative for both serum HBsAg and anti-HCV antibody. A panel of six human HCC cell lines (Hep 3B, HA22T, HCC36, Tong, SK-Hep-1 and HuH-7) and two human hepatoblastoma cell lines (Hep G2 and HuH-6), were included in this study (Doi, 1976; Fogh et al, 1977; Aden et al, 1979; Nakabayashi et al, 1982).

### Microsatellite polymorphism analysis

To detect allelic losses, a PCR-based approach as described by Kastury et al was performed (1996). Two loci, D3S1300 (within the *FHIT* gene) and D3S1312 (centromerically flanking the *FHIT* gene) were selected (Druck et al, 1995; Kastury et al, 1996; Ohta et al, 1996; Sozzi et al, 1996*a*).

**Table 1** Aberrant transcripts observed in HCC

| Case | Age (years) | Sex | HBsAg | HCV | Cirrhosis | Abnormal transcript | | LOH |
|---|---|---|---|---|---|---|---|---|
| | | | | | | Non-tumour | Tumour | |
| 1 | 77 | M | + | – | – | Ex 4–8 loss | Ex 4–8 loss | – |
| 2 | 67 | M | – | + | – | – | Ex 4–7 loss with Alu insertion | – |
| 3 | 74 | F | – | + | + | Ex 4–8 loss | Ex 5–8 loss | – |
| 4 | 37 | M | + | – | – | – | – | – |
| 5 | 53 | M | + | – | + | Ex 4–8 loss | Ex 5–7 loss | – |
| 6 | 57 | M | + | – | – | – | – | – |
| 7 | 76 | F | – | + | + | Ex 5–7 loss | – | – |
| 8 | 51 | M | + | – | + | Ex 4–6 loss | Ex 4–6 loss | – |
| 9 | 62 | M | + | – | + | Ex 4–6 loss | Ex 4–6 loss | – |
| 10 | 60 | F | + | – | + | Ex 4–6 loss | Ex 5–7 loss | – |
| 11 | 20 | M | + | – | – | – | Ex 4–8 loss | – |
| 12 | 47 | F | + | – | – | – | – | N |
| 13 | 71 | F | – | + | + | – | Ex 5–8 loss | N |
| 14 | 50 | M | – | + | + | Ex 4–8 loss | – | – |
| 15 | 62 | M | + | + | + | – | Ex 5–7 loss | – |
| 16 | 67 | F | – | + | + | – | – | – |
| 17 | 72 | M | + | – | + | – | Ex 4–8 loss | N |
| 18 | 83 | M | – | + | + | – | Ex 4–8 loss | – |

In addition to the aberrant transcripts indicated above, a normal-sized transcript was present in all the tumorous and non-tumorous tissues examined except in the tumorous tissue of case 1. Loci analysed for LOH were D3S1300 and D3S1312. HBsAg, hepatitis B virus surface antigen; HCV, anti-hepatitis C virus antibody; Cir, cirrhosis; LOH, loss of heterozygosity; Ex, exon; M, male; F, female; Alu, Alu repeat; N, not informative.

### RNA extraction, reverse transcriptase-polymerase chain reaction (RT-PCR) and sequence analysis

The transcripts of the *FHIT* gene were examined using RT-PCR. RNA was purified from tumorous and non-tumorous liver tissues and cell lines, and cDNA was generated from RNA as described previously (Chen et al, 1997). Nested PCR amplifications were carried out using primers, flanking the full coding sequence of the *FHIT* cDNA, as described previously (Ohta et al, 1996). All the reactions were performed at least twice and for verification of the integrity of the RNA samples, control RT-PCR amplifications using primers specific for glyceraldehyde 3-phosphate dehydrogenase (G3PDH) gene were performed (Nobori et al, 1994). Procedures for sequencing of PCR products have been described previously (Chen et al, 1997).

### RESULTS AND DISCUSSION

To study abnormalities of the *FHIT* gene, we first examined chromosome 3p14.2 by microsatellite polymorphism analysis. A total of 15 out of 18 (83.3%) cases were informative for at least one of the two loci examined and none of them revealed LOH (data not shown). Although small deletions and rearrangements could not be excluded, the results suggest that the chromosome 3p14.2 region as defined by markers, D3S1300 and D3S1312 was intact in both alleles in these cases.

The expressions of the *FHIT* transcripts were then examined by RT-PCR amplification. Except in the tumorous tissue of case 1, normal-sized bands were detected readily in all the tissues and the cell lines that we examined. However, faint bands of smaller size were also seen in 12 out of the 18 tumorous (66.7%) and 8 out of the 18 non-tumorous (44.4%) liver tissues ($P > 0.05$, McNemar's test, $\chi^2 = 1.12$, d.f. = 1). In addition, five out of the eight liver tumour cell lines also revealed abnormally smaller bands.

**Figure 1** A schematic representation of the aberrant transcripts detected in the HCC specimens and liver cancer cell lines. The coding exons of the *FHIT* gene are in grey colors and a segment of Alu repeat is indicated by oblique lines. Numbers in the bars, *FHIT* exons; arrowheads, abnormal junctions between exons 4 and 8, exons 4 and 9, exons 3 and 7, exons 3 and 9, and exon 3, Alu repeat and exon 8; T, tumorous; and N, non-tumorous liver tissues; numbers following T or N are case numbers. ■, Coding exons; □, non-coding exons; ▨, Alu sequence

Subsequently, the RT-PCR products were examined by sequence analysis. For the normal-sized products, no variations were detected. However, sequence analysis of the smaller bands revealed that they represented aberrant *FHIT* transcripts. A detailed description of these aberrant transcripts is summarized in Figure 1 and Table 1. In brief, five different types of aberrant transcripts with loss of various exons from 4–8 were observed: products lacking exons 5–7, 5–8, 4–6, 4–8 and a product lacking exons

 © Cancer Research Campaign 1998

4–7 and an insertion of a 138-bp Alu sequence. As the fusion functions all coincided exactly with the splice sites, these aberrant transcripts might represent alternatively spliced products.

Six cases (cases 1, 3, 5, 8, 9 and 10) revealed aberrant transcripts in both tumorous and non-tumorous liver tissues. Three of them had the same abnormal pattern of the aberrant transcripts between tumorous and non-tumorous tissues; however, in the other three cases (cases 3, 5 and 10), the patterns were different between the paired samples (Table 1). Whereas six cases had aberrant transcripts only in their tumorous tissues, we also found two cases (cases 7 and 14) showing an aberrant transcript in their non-tumorous liver tissues only (Table 1).

Taken together, our results suggested that abnormalities of the *FHIT* gene might not be related to hepatocarcinogenesis. First, although in agreement with previous reports of other human malignancies (Negrini et al, 1996; Ohta et al, 1996; Sozzi et al, 1996*a,b*), aberrant transcripts of the *FHIT* gene were frequently detected in the tumorous liver tissues, they also existed in the non-tumorous liver tissues. Recently, Boldog et al reported the presence of the aberrant *FHIT* transcripts in the normal fetal brain cDNA and concluded that the alternative splicing definitely occurs in normal human tissues (Boldog et al, 1997). This is consistent with our findings and may explain the currently and most previously reported abnormal *FHIT* transcripts. Therefore, it is possible that the *FHIT* gene is simply located near to but is not the true 'target' that drives a clonal selection process (Thiagalingam et al, 1996). Second, in 17 out of the 18 tumorous liver tissues examined in this study, a normal-sized RT-PCR product, containing the complete coding region of the *FHIT* gene, was observed. In addition, sequence analysis revealed that they were all intact. Ohta et al. (1996) also detected full-length RT-PCR products in nearly all cases showing aberrant transcripts but suggested that these might have resulted from the presence of contaminating non-neoplastic cells. However, because the normal RT-PCR products were always present at a robust level, and the normal products were also observed in all eight liver tumour cell lines, we believe that these normal products are derived from neoplastic cells. Third, according to the Knudson's two-hit model for inherited and sporadic forms of retinoblastoma, tumour-suppressor genes can usually be inactivated by deletion of one allele by various mechanisms, and inactivation of another allele by mutation, loss or other mechanisms (Knudson, 1971; Lasko et al, 1991). Reviewing the literature, in HCC allele losses have been documented on chromosomes 1, 4, 5, 8, 10, 11, 13, 16, 17 and 22; however, no such changes have ever been reported on chromosome 3 (Ding and Habib, 1995). Sozzi et al (1996a) reported a 92% and a 63% loss of one *FHIT* allele at loci D3S1300 and D3S1312 in primary lung tumours respectively, but, in this study, we did not detect any allelic loss at either loci in any of the HCC tissues that we examined. Therefore, although more loci should be studied to define the possible small deletions and rearrangements, we believe that the *FHIT* gene might not be involved causally in hepatocarcinogenesis. Fourth, the Fhit protein is a typical dinucleoside 5′,5‴-$P^1$, $P^3$-triphosphate ($Ap_3A$) hydrolase that catalyses the hydrolysis of dinucleoside polyphosphates with $Ap_3A$ as the preferred substrate (Huang et al, 1995; Barnes et al, 1996; Druck et al, 1997). Although $Ap_3A$ has potential significance in the response to different kinds of stress, blood platelet function, vasomotor activity and hepatic function control (Bernet et al, 1991), no strong evidence has been shown that it may contribute to carcinogenesis (Barnes et al, 1996).

HCC has been shown to be closely related to chronic HBV or HCV infection and cirrhosis has been recognized as a precancerous lesion (Beasley et al, 1981; Okuda, 1992; Wright et al, 1992; Chen, 1993). In this study, irrespective of HBV or HCV infection, the non-tumorous liver tissues contained a fairly high frequency (44.4%) of abnormal *FHIT* transcripts. The *FHIT* gene, containing a common fragile region, FRA3B, is susceptible to the breakage caused by physical or chemical carcinogens (Paradee et al, 1995). As liver plays a major role in the metabolism of most drugs or toxins, and several carcinogens, such as 2,3,7,8-tetrachlorodibenzo-p-dioxin, have been proved to regulate the expression of a number of genes involved in cell growth control by activities on their promoters (Lee et al, 1996), it is possible that similar effects could result in the high frequency of change in the *FHIT* gene in the chronically damaged (tumorous or cirrhotic) liver tissues.

In conclusion, these studies indicate that abnormalities of the *FHIT* gene transcripts occur in a fairly high proportion of tumorous and non-tumorous liver tissues. However, it might not be causally related to the hepatocarcinogenesis. To clarify further the molecular basis of *FHIT* fragility and its involvement in human cancers, additional evaluations of tumours and normal tissues, as well as complete sequencing of the specific breakpoints, are needed.

### ACKNOWLEDGEMENT

This work was supported in part by a research grant from the Taipei Municipal Jen-Ai Hospital.

### REFERENCES

Aden D, Fogel A, Plotkin S, Damjanov I and Knowles B (1979) Controlled synthesis of HBsAg in a differentiated human liver carcinoma-derived cell line. *Nature* **282**: 615–618

Barnes LD, Garrison PN, Siprashvili Z, Guranowski A, Robinson AK, Ingram SW, Croce CM, Ohta M and Huebner K (1996) Fhit, a putative tumor suppressor in humans, is a dinucleoside 5′,5‴-$P^1$, $P^3$ triphosphate hydrolase. *Biochemistry* **35**: 11529–11535

Beasley RP, Hwang L-Y, Lin C-C and Chien C-S (1981) Hepatocellular carcinoma and hepatitis B virus: a prospective study of 22,707 men in Taiwan. *Lancet* **2**: 1129–1133

Bernet D, Pinto RM, Sillero A and Cameselle JC (1991) Location of dinucleoside triphosphatase in the matrix space of rat liver mitochondria. *FEBS Lett* **232**: 286–288

Boldog F, Gemmill RM, West J, Robinson M, Robinson L, Li E, Roche J, Todd S, Waggoner B, Lundstrom R, Jacobson J, Mullokandov MR, Klinger H and Krabkin HA (1997) Chromosome 3p14 homozygous deletions and sequence analysis of FRA3B. *Hum Mol Genet* **6**: 193–203

Chen D-S (1993) From hepatitis to hepatoma; lessons from type B hepatitis. *Science* **262**: 369–370

Chen Y-J, Chang J-G, Shih L-S, Chen P-H, Endo M, Whang-Peng J and Chen Y-MA (1997) Frequent detection of aberrant RNA transcripts of the *CDKN2* gene in human gastric adenocarcinoma. *Int J Cancer* **71**: 350–354

Cohen AJ, Li FP, Berg S, Marchetto DJ, Tsai S, Jacobs SC and Brown RS (1979) Hereditary renal-cell carcinoma associated with a chromosomal translocation. *N Engl J Med* **301**: 592–595

Ding S-F and Habib NA (1994) Malignant tumours of the liver and biliary system. In *Cancer: a Molecular Approach*. Lemoine NR, Cooke T and Neoptolenos J (ed.), pp. 95–105. Blackwell Scientific Publications: Oxford

Ding S-F and Habib NA (1995) Loss of heterozygosity in liver tumours. *J Hepatol* **22**: 230–238

Doi I (1976) Establishment of a cell line and its clonal sublines from a patient with hepatoblastoma. *Gann* **67**: 1–10

Druck T, Kastury K, Hadaczek P, Podolski J, Toloczko A, Sikorski A, Ohta M, LaForgia S, Lasota J, McCue P, Lubinski J and Huebner K (1995) Loss of

© Cancer Research Campaign 1998

heterozygosity at the familial RCCt (3;8) locus in most clear cell renal carcinoma. *Cancer Res* **55**: 5348–5355

Druck T, Hadaczek P, Fu T-B, Ohta M, Siprashvili Z, Baffa R, Negrini M, Kastury K, Veronese ML, Rosen D, Rothstein J, McCue P, Cotticelli MG, Inoue H, Croce CM and Huebner K (1997) Structure and expression of the human *FHIT* gene in normal and tumor cells. *Cancer Res* **57**: 504–512

Fogh J, Wright WC and Loveless JD (1977) Absence of HeLa cell contamination in 169 cell lines derived from human tumours. *J Natl Cancer Inst* **58**: 209–214

Huang Y, Garrison PN and Barnes LD (1995) Cloning of the *Schizosaccharomyces pombe* gene encoding diadenosine 5′,5‴-p-1,p-4-tetraphosphate [ap(4)a] asymmetrical hydrolase: sequence similarity with the histidine triad (HIT) protein family. *Biochem J* **312**: 925–932

Kastury K, Baffa R, Druck T, Ohta M, Cotticelli MG, Inoue H, Negrini M, Rugge M, Huang D, Croce CM, Palazzo J and Huebner K (1996) Potential gastrointestinal tumor suppressor locus at the 3p14.2 FRA3B site identified by homozygous deletions in tumor cell lines. *Cancer Res* **56**: 978–983

Knudson AG Jr (1971) Mutation and cancer: statistical study of retinoblastoma. *Proc Natl Acad Sci USA* **68**: 820–823

Lasko D, Cavenee W and Nordenskjold M (1991) Loss of constitutional heterozygosity in human cancer. *Annu Rev Genet* **25**: 281–314

Lee DC, Barlow KD and Gaido KW (1996) The actions of 2,3,7,8-tetrachlorodibenzo-p-dioxin on transforming growth factor-$\beta_2$ promoter activity are localized to the TATA box binding region and controlled through a tyrosine kinase-dependent pathway. *Toxicol Appl Pharmacol* **137**: 90–99

Nakabayashi H, Taketa K, Miyano K, Yamane Y and Sato J (1982) Growth of human hepatoma cell lines with differentiated functions in chemically defined medium. *Cancer Res* **42**: 3858–3863

Negrini M, Monaco C, Vorechovsky I, Ohta M, Druck T, Baffa R, Huebner K and Croce CM (1996) The *FHIT* gene at 3p14.2 is abnormal in breast carcinomas. *Cancer Res* **56**: 3173–3179

Nobori T, Miura K, Wu DJ, Lois A, Takabayashi K and Carson DA (1994) Deletions of the cyclin-dependent kinase-4 inhibitor gene in multiple human cancers. *Nature* **368**: 753–756

Ohta M, Inoue H, Cotticelli MG, Kastury K, Baffa R, Palazzo J, Siprashvili Z, Mori M, McCue P, Druck T, Croce CM and Huebner K (1996) The human *FHIT* gene, spanning the chromosome 3p14.2 fragile site and renal carcinoma associated translocation breakpoint, is abnormal in digestive tract cancers. *Cell* **84**: 587–597

Okuda K (1992) Hepatocellular carcinoma: recent progress. *Hepatology* **15**: 948–963

Paradee W, Mullins C, He Z, Glover T, Wilke C, Opalka B, Schutte J and Smith D (1995) Precise localization of aphidicolin-induced breakpoints on the short arm of human chromosome 3. *Genomics* **27**: 358–361

Sozzi G, Veronese ML, Negrini M, Baffa R, Cotticelli MG, Inoue H, Tornielli S, Pilotti S, Gregorio LD, Pastorino U, Pierotti MA, Ohta M, Huebner K and Croce CM (1996*a*) The *FHIT* gene at 3p14.2 is abnormal in lung cancer. *Cell* **85**: 17–26

Sozzi G, Alder H, Torkielli S, Corletto V, Baffa R, Veronese ML, Negrini M, Pilotti S, Pierotti MA, Huebner K and Croce CM (1996*b*) Aberrant *FHIT* transcripts in merkel cell carcinoma. *Cancer Res* **56**: 2472–2474

Sugimura T (1992) Multistep carcinogenesis: a 1992 perspective. *Science* **258**: 603–607

Thiagalingam S, Lisitsyn NA, Hamaguchi M, Wigler MH, Willson JKV, Markowitz SD, Leach FS, Kinzler KW and Vogelstein B (1996) Evaluation of the *FHIT* gene in colorectal cancers. *Cancer Res* **56**: 2936–2939

Wright TL, Venook AP and Millward-Sadler GH (1992) Hepatic tumours. In *Wright's Liver and Biliary Disease*. Millward-Sadler GH, Wright R and Arthur MJP (eds), pp. 1079–1121. WB Saunders: London

© Cancer Research Campaign 1998